# Supplementary material for: Climate predicts geographic and temporal variation in mosquito-borne disease dynamics on two continents
Source: Nat Commun. 2021 Feb 23;12:1233. doi: 10.1038/s41467-021-21496-7 (PMC7902664; doi:10.1038/s41467-021-21496-7)
Supplement: Supplementary file 1 — Supplementary Information [file 41467_2021_21496_MOESM1_ESM.pdf]

Supplementary information for "Climate predicts geographic and temporal variation in mosquito-borne disease dynamics on two continents"

Supplementary Table 1: Effect of temperature on dengue transmission dynamics from prior studies. This table provides information used in Figure 5 of the main text to visualize the relationship between temperature and dengue risk compared with the relative basic reproductive number derived from the trait-based model. Asterisks indicate that the mean temperature was calculated as the average value of the minimum and maximum temperatures provided in the text (measured or estimated from temperature plots). Coefficient values are the values calculated in regression models in the sourced references, they indicate the estimated effect on dengue cases given a one-unit change in the temperature metric.

Study location	Dengue metric	Mean temperature	Temperature metric	Temperature metric time lag	Coefficient value	Source
Mexico	Weekly cases	14*	Weekly minimum	None	0.44	[1]
Mexico	Weekly cases	15*	Weekly minimum	None	0.58	[1]
Mexico	Monthly cases	16.5*	Monthly minimum	4-8 weeks	0.03-0.15	[2]
China	Monthly cases	19.5	Minimum	4 weeks	0.732	[3]
Guadeloupe	Yearly or seasonal cases	22*	Minimum	5 weeks	0.108	[4]
Vietnam	Monthly cases	22.5*	Monthly mean	8 weeks	0.23	[5]
Thailand	Monthly cases	23	Monthly minimum	None	0.99	[6]
Bangladesh	Monthly cases	23*	Mean	4 weeks	6.07	[7]
Mexico	Weekly incidence per 100,000 people	25	Mean weekly Sea Surface Temperature	5 weeks	0.2	[8]
Guadeloupe	Yearly or seasonal cases	26.5*	Mean	11 weeks	0.228	[4]
Taiwan	Monthly cases	29*	Deviation between monthly maximum and mean	None	-0.126	[9]
Bangladesh	Monthly cases	30*	Monthly maximum	None	0.0105	[10]
Vietnam	Monthly cases	33.5*	Monthly mean	8 weeks	-1.94	[5]
Colombia	Monthly or Epi-period cases	32	Monthly mean	None	0.001	[11]
Thailand	Monthly cases	35*	Monthly maximum	4 weeks	-0.609	[12]

Supplementary Table 2: Number of days with interpolated temperature, rainfall, and humidity by site within study period. There were 1,918 days in total for sites within Ecuador and 2,069 days in total for sites within Kenya. Interpolated rainfall values indicate days where there was one or more missing days with a rainfall value in the prior 14 days (inclusive of measurement date).

Country	Site	Days with interpolated temperature	Days with interpolated monthly rainfall	Days with interpolated humidity
Ecuador	Huaquillas	331	443	331
Ecuador	Machala	6	78	306
Ecuador	Portovelo	490	601	489
Ecuador	Zaruma	974	434	322
Kenya	Chulaimbo	289	511	288
Kenya	Kisumu	325	506	305
Kenya	Msambweni	142	602	142
Kenya	Ukunda	443	623	867

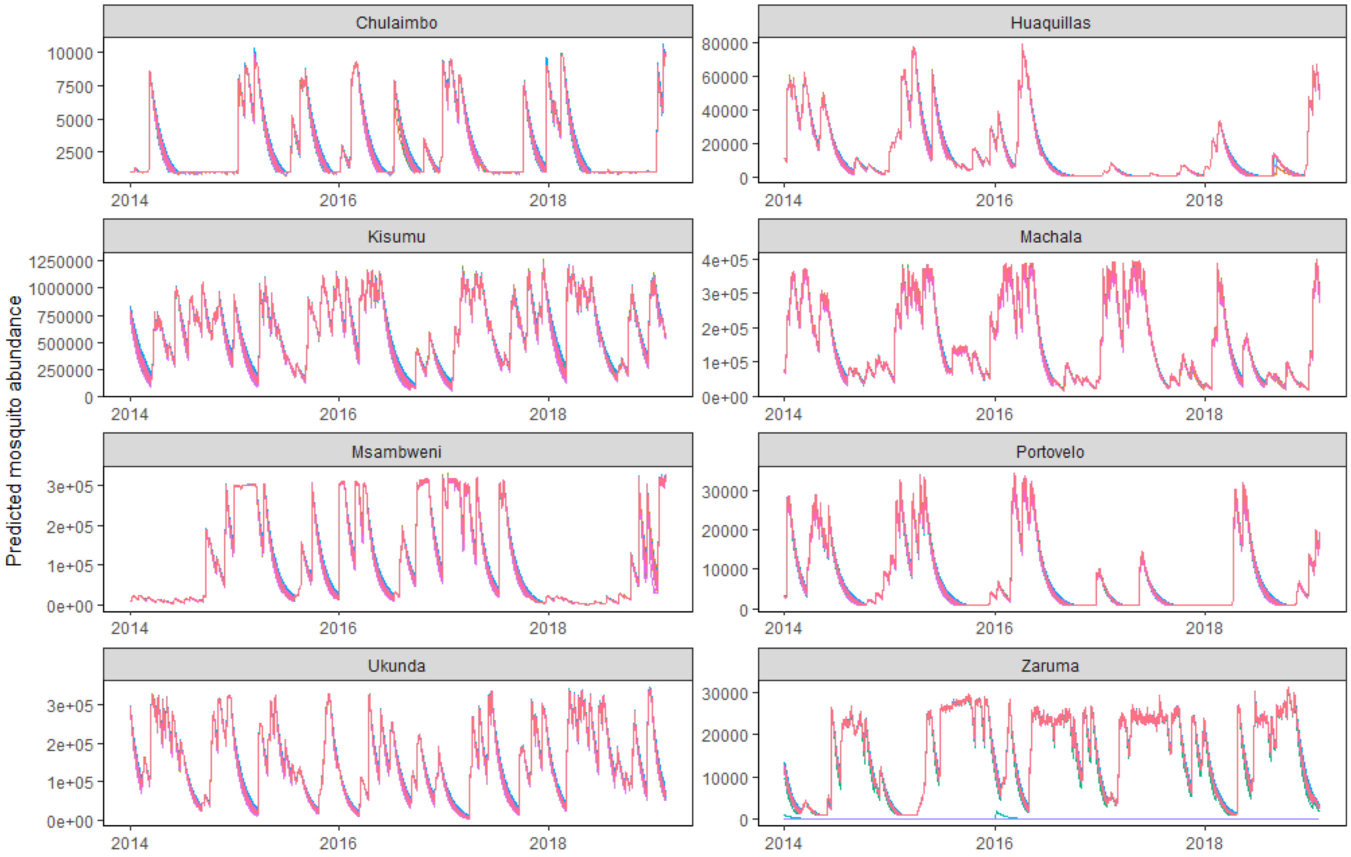

Supplementary Figure 1: Mosquito predictions are not affected by critical thermal minimum, maximum, and rate constants used in *Aedes aegypti* life history traits. To determine the extent to which variations in *Aedes aegypti* life history traits affect model predictions, we conducted a sensitivity analysis. For the sensitivity analysis, we randomly sampled 50 different c, T_0, and T_m estimates for temperature-dependent traits from the posterior distributions found in [13] and ran the SEI-SEIR model with those estimates. This plot shows the total predicted mosquitoes (N_m) for each site through time. The results for each model run are shown as a different colored line.

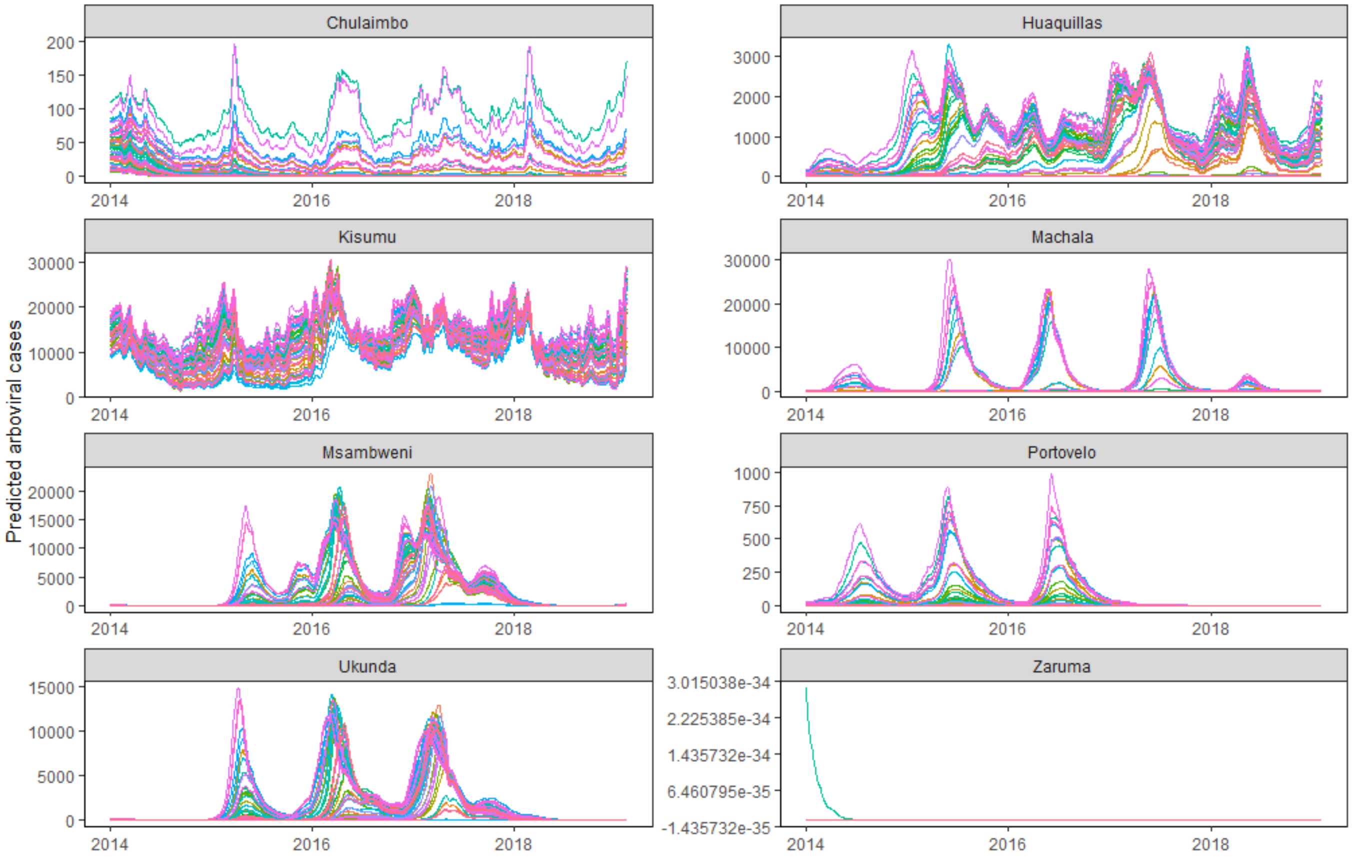

Supplementary Figure 2: Trajectories of predicted arboviral cases are not affected by critical thermal minimum, maximum, and rate constants used in *Aedes aegypti* life history traits. To determine the extent to which variations in *Aedes aegypti* life history traits affected model predictions, we conducted a sensitivity analysis. For the sensitivity analysis, we randomly sampled 50 different c, T_0, and T_m estimates for temperature-dependent traits from the posterior distributions found in [13] and ran the SEI-SEIR model with those estimates (same models as shown in Figure S1). This plot shows predicted cases (I_h) for each site through time. The results for each model run are shown as a different colored line. The different trait values varied the magnitude of predicted cases in many settings, but not the temporal dynamics. Many simulations overlap for Portovelo and Zaruma.

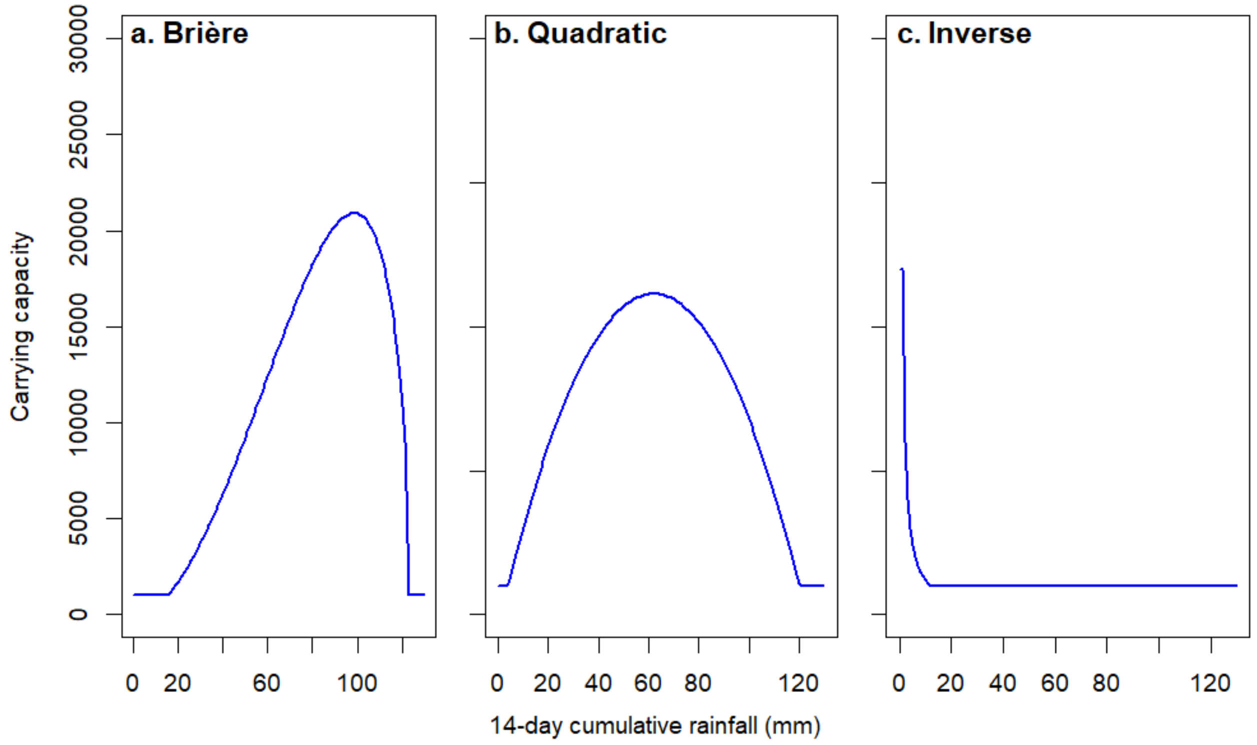

Supplementary Figure 3: Hypothesized functional forms for effects of rainfall on mosquito carrying capacity. We tested three hypothesized functional relationships between 14-day cumulative rainfall values (following [14]) and mosquito carrying capacity: (a) Brière, in which carrying capacity increases with increasing rainfall until a threshold where flushing occurs; (b) quadratic, in which carrying capacity peaks at intermediate rainfall values, similar to [15]; and (c) inverse, in which mosquito abundance is greatest during periods of drought, similar to [14]. In these plots, temperature, maximum rainfall, and the human population are held constant at 29°C, 123 mm, and 20,000 people, respectively. See Methods for functional form equations.

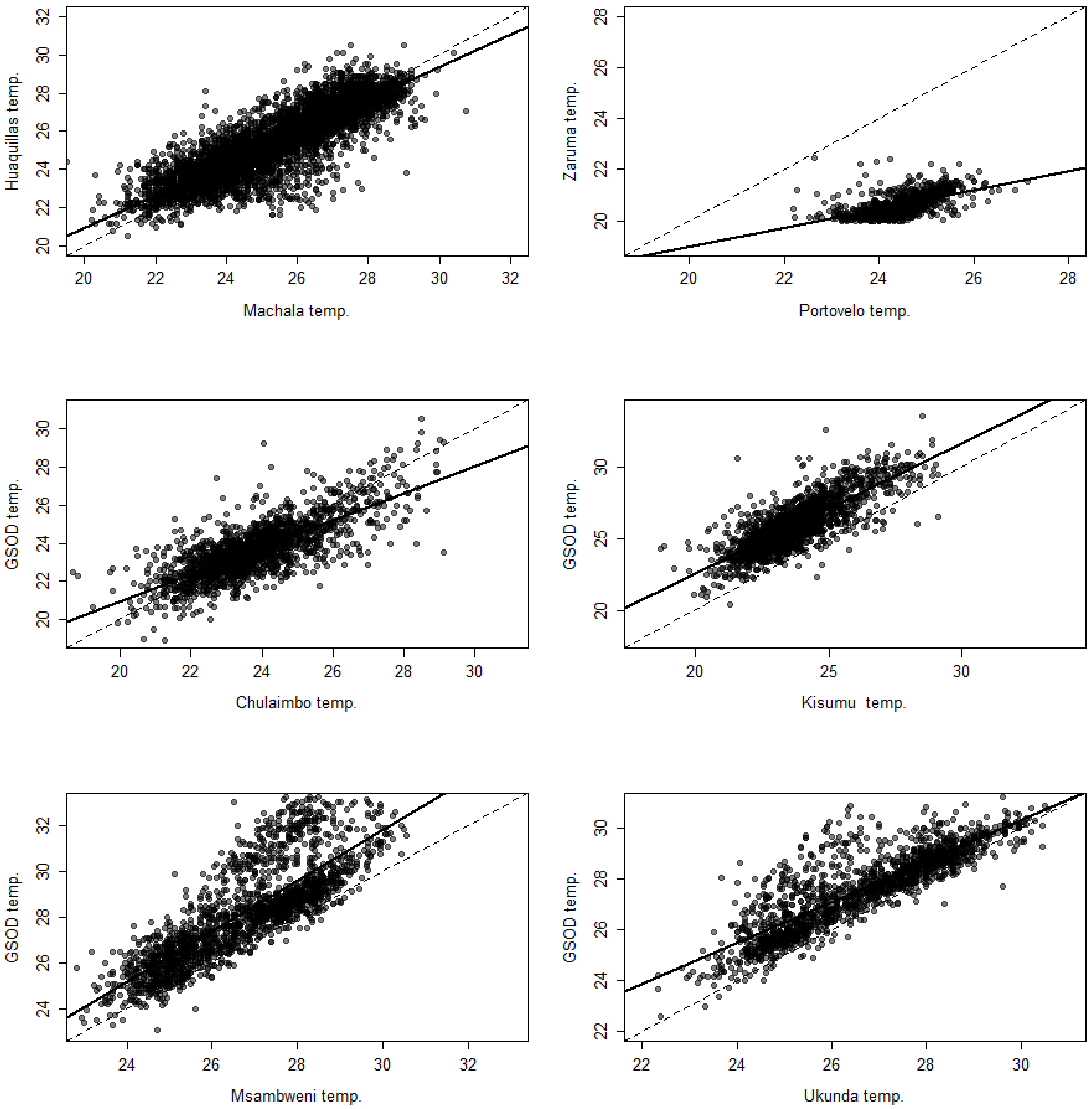

Supplementary Figure 4: Scatterplot comparing ambient temperatures measured at study sites with datasets used for interpolation. For Ecuador, we used the nearest study site values when possible or else the long term mean temperature values for the corresponding Julien day. For Kenya, we used NOAA Global Surface Summary of the Day datasets from Kisumu Airport for Kisumu and Chulaimbo and from Mombasa Airport for Msambweni and Ukunda. Dashed lines indicate the y = x line and solid lines indicate the linear regression line used to interpolate data.

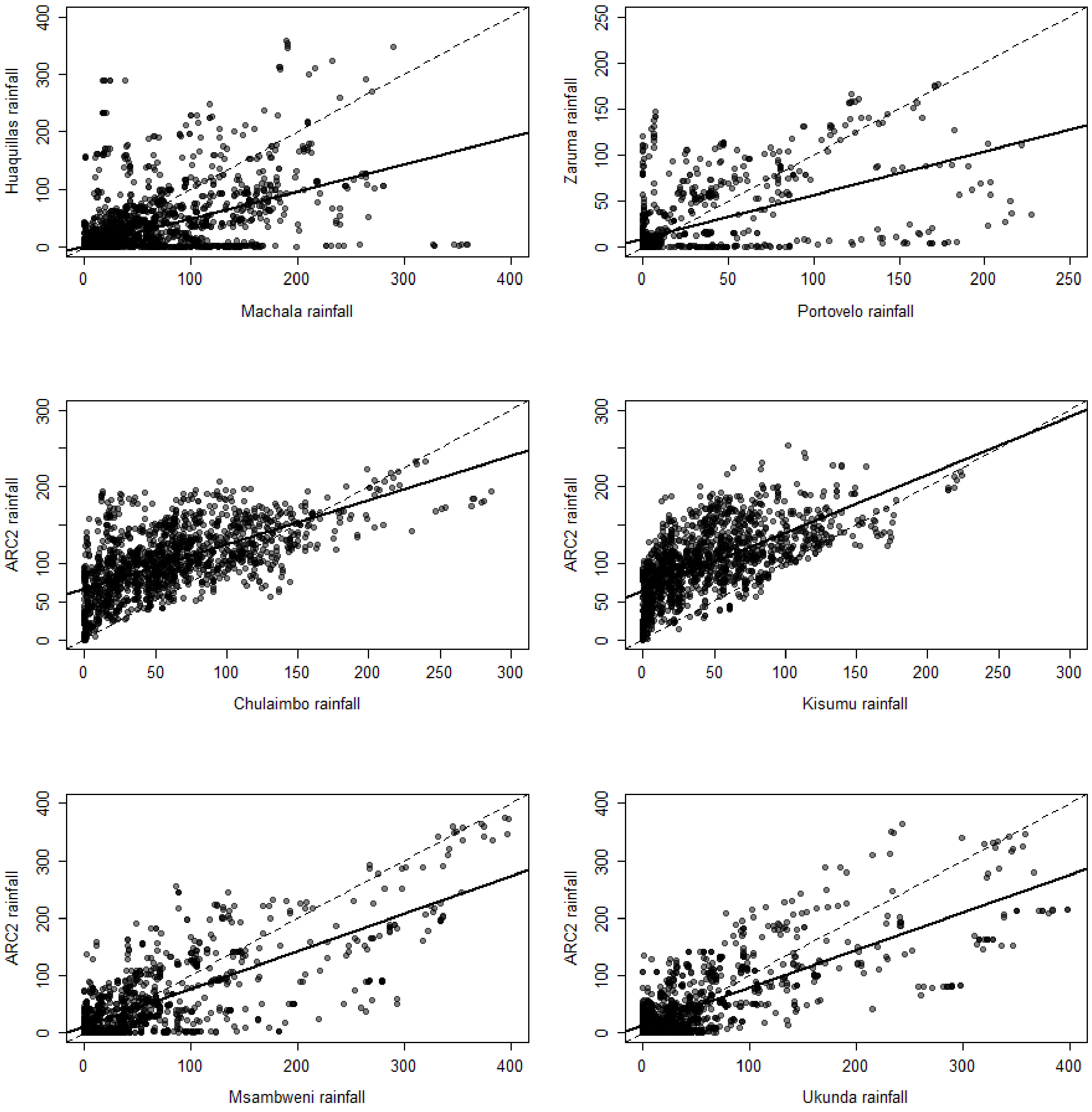

Supplementary Figure 5: Scatterplot comparing daily values for cumulative rainfall in the prior 14 days measured at study sites with datasets used for interpolation. For Ecuador, we used the nearest study site values when possible or else the long term mean 14-day cumulative rainfall values for the corresponding Julien day. For Kenya, we used NOAA Global Surface Summary of the Day datasets from Kisumu Airport for Kisumu and Chulaimbo and from Mombasa Airport for Msambweni and Ukunda. Dashed lines indicate the y = x line and solid lines indicate the linear regression line used to interpolate data.

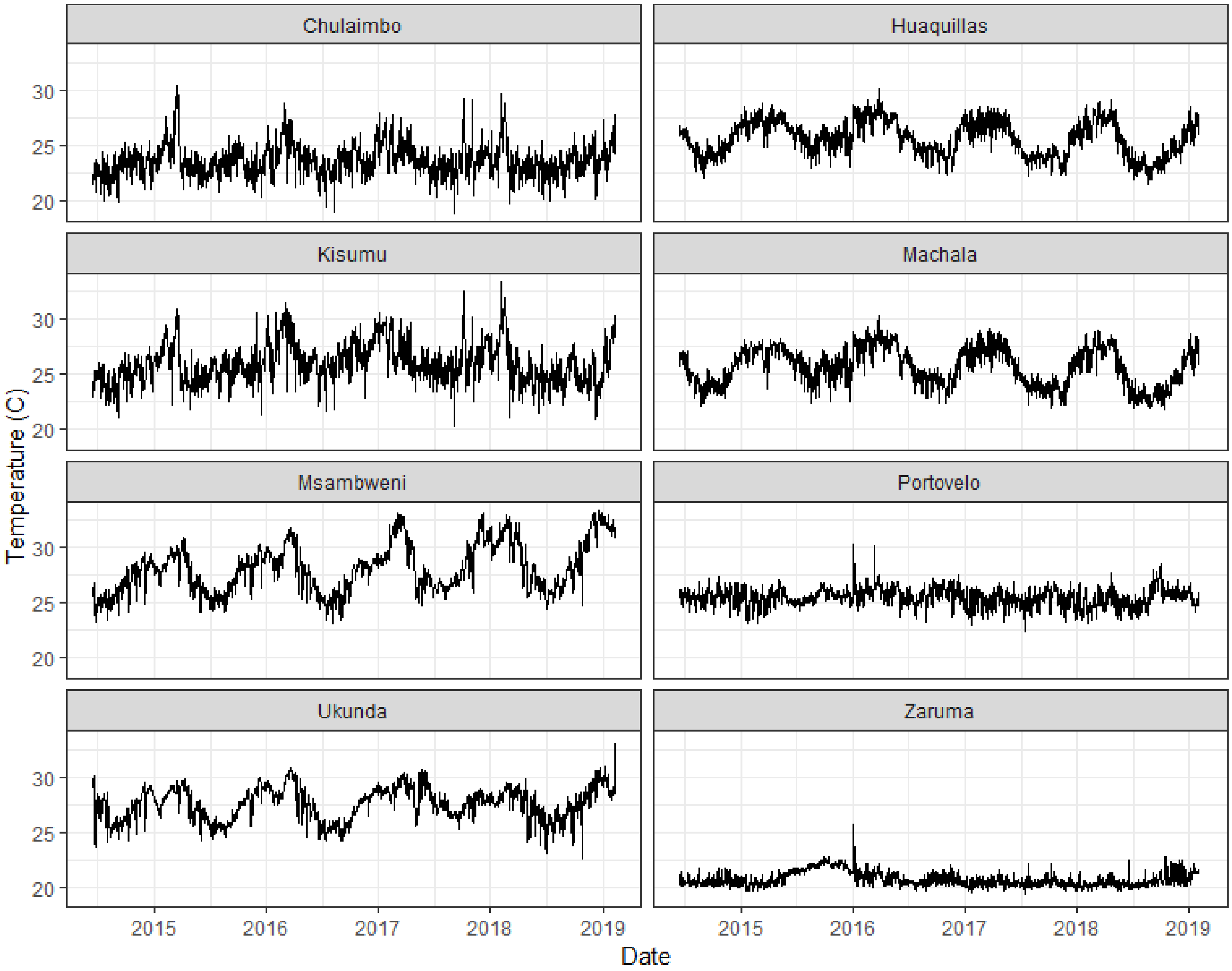

Supplementary Figure 6: Daily temperatures across sites within study period. Kenya sites are on the left and Ecuador sites are on the right.

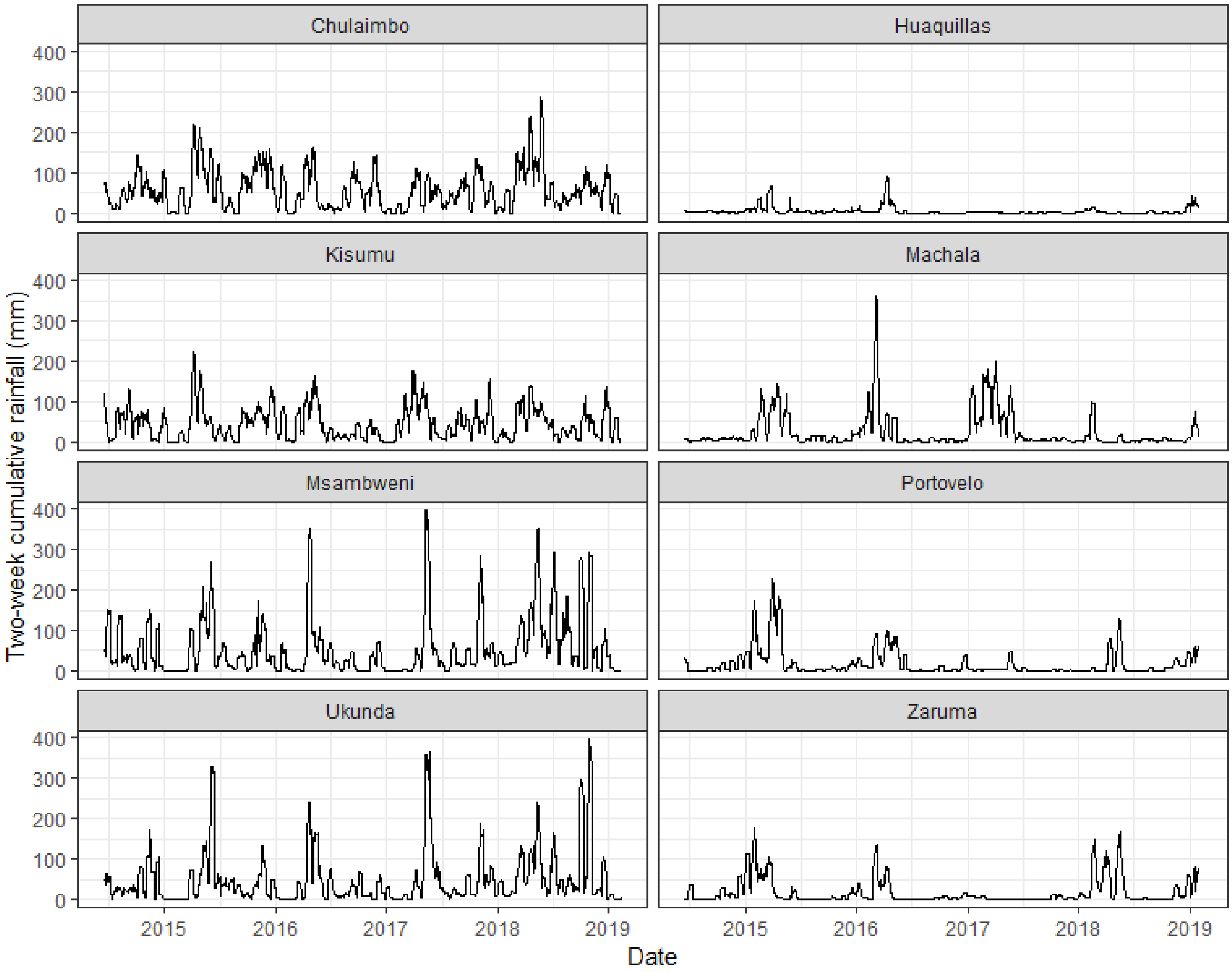

Supplementary Figure 7: Daily values of cumulative 14-day rainfall across sites within study period. Kenya sites are on the left and Ecuador sites are on the right.

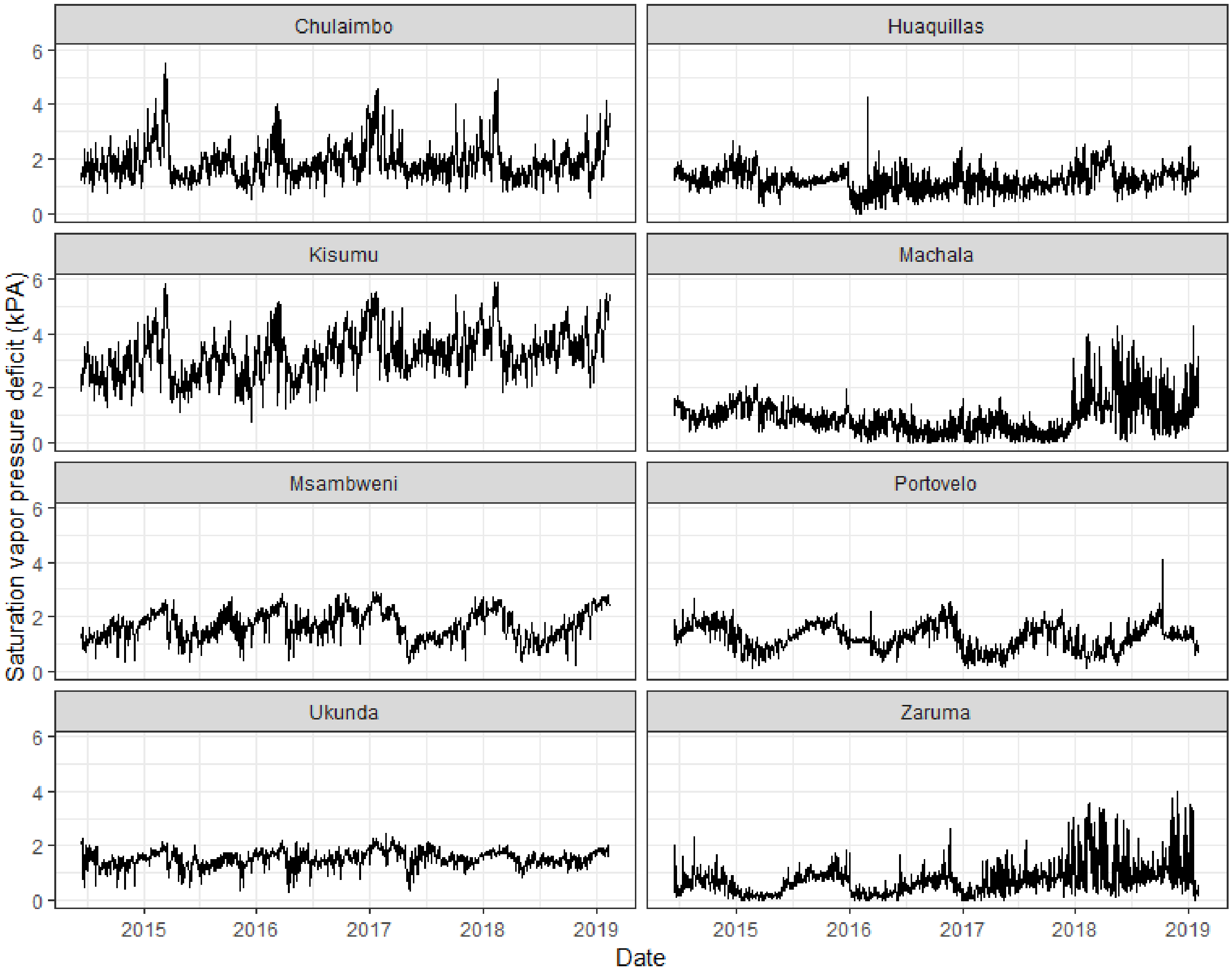

Supplementary Figure 8: Daily saturation vapor pressure deficit across sites within study period. Kenya sites are on the left and Ecuador sites are on the right.

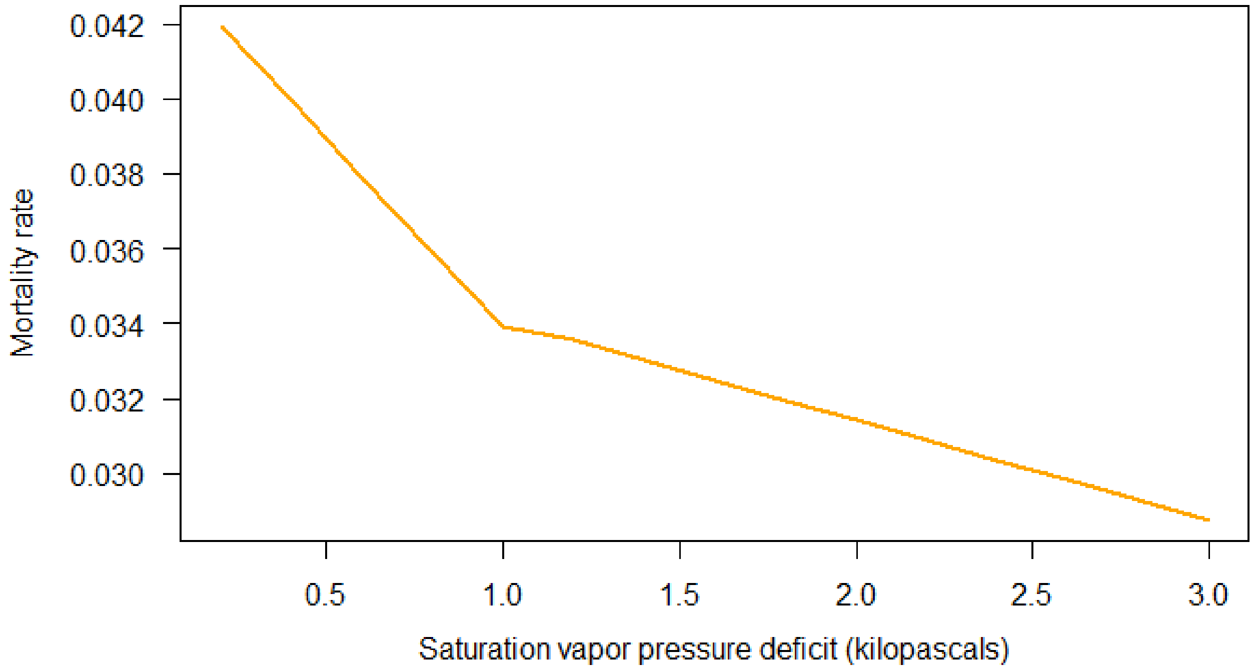

Supplementary Figure 9: Mosquito mortality rate as a function of saturation vapor pressure deficit (SVPD). This relationship is a step function where the slope of the linear relationship is steeper for SVPD ≤ 1 compared with SVPD > 1. The step function is also scaled differently for SVPD ≤ 1 and SVPD > 1 to restrict the mortality rate within the range of mortality rates observed in other studies; these scaling factors make the function appear nonlinear between 1.0 and 1.2 in the plot.

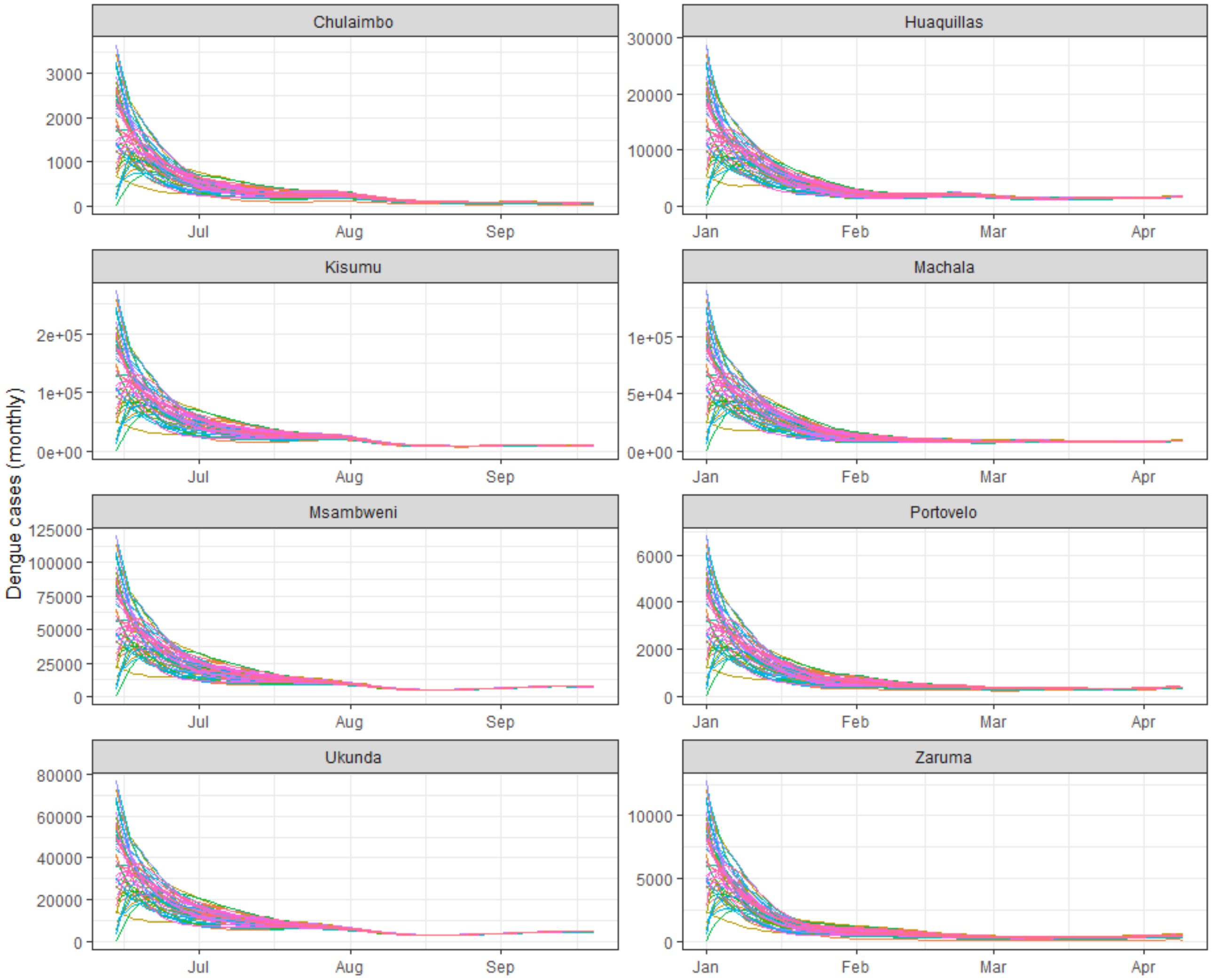

Supplementary Figure 10: Models converge after ~90 days regardless of initial conditions. To determine how the model's initial conditions affected the magnitude and trajectories of each compartment and across sites, we conducted a sensitivity analysis. We randomly sampled 50 different proportions of starting conditions for each compartment where $S_V + E_V + I_V = 1$ and $S_H + E_H + I_H + R_H = 1$ using Latin hypercube sampling. Latin hypercube sampling is a statistical method for generating a random sample of values from a multidimensional distribution. We used the optimumLHS function in the lhs package in R to generate the random sample of initial proportions for each compartment. This plot shows the initial trajectories of predicted cases for each study site. All other model compartments also converged after ~90 days.

Supplementary References:

1. Hurtado-Daz M, Riojas-Rodrguez H, Rothenberg S, Gomez-Dantes H, Cifuentes E. Impact of climate variability on the incidence of dengue in Mexico. Trop Med Int Heal. 2007;12. doi:10.1111/j.1365-3156.2007.01930.x
2. Colón-González FJ, Bentham G, Lake IR. Climate Variability and Dengue Fever in Warm and Humid Mexico. Am J Trop Med Hyg. 2011;84: 757–763. doi:10.4269/ajtmh.2011.10-0609
3. Wang C, Jiang B, Fan J, Wang F, Liu Q. A Study of the Dengue Epidemic and Meteorological Factors in Guangzhou, China, by Using a Zero-Inflated Poisson Regression Model. Asia Pacific J Public Heal. 2014;26: 48–57. doi:10.1177/1010539513490195
4. Gharbi M, Quenel P, Gustave J, Cassadou S, Ruche G La, Girdary L, et al. Time series analysis of dengue incidence in Guadeloupe, French West Indies: Forecasting models using climate variables as predictors. BMC Infect Dis. 2011;11: 166. doi:10.1186/1471-2334-11-166
5. Minh An DT, Rocklöv J. Epidemiology of dengue fever in Hanoi from 2002 to 2010 and its meteorological determinants. Glob Health Action. 2014;7: 23074. doi:10.3402/gha.v7.23074
6. Sriprom M, Chalvet-Monfray K, Chaimane T, Vongsawat K, Bicout DJ. Monthly district level risk of dengue occurrences in Sakon Nakhon Province, Thailand. Sci Total Environ. 2010;408: 5521–5528. doi:10.1016/J.SCITOTENV.2010.08.024
7. Sharmin S, Glass K, Viennet E, Harley D. Interaction of Mean Temperature and Daily Fluctuation Influences Dengue Incidence in Dhaka, Bangladesh. Kasper M, editor. PLoS Negl Trop Dis. 2015;9: e0003901. doi:10.1371/journal.pntd.0003901
8. Laureano-Rosario AE, Garcia-Rejon JE, Gomez-Carro S, Farfan-Ale JA, Muller-Kargera FE. Modelling dengue fever risk in the State of Yucatan, Mexico using regional-scale satellite-derived sea surface temperature. Acta Trop. 2017;172: 50–57. doi:10.1016/j.actatropica.2017.04.017
9. Wu P-C, Guoa H-R, Lung S-C, Lin C-Y, Su H-J. Weather as an effective predictor for occurrence of dengue fever in Taiwan. Acta Trop. 2007;103: 50–57. doi:10.1016/j.actatropica.2007.05.014

10. Karim MN, Munshi SU, Anwar N, Alam MS. Climatic factors influencing dengue cases in Dhaka city: a model for dengue prediction. Indian J Med Res. 2012;136: 32–9. Available: http://www.ncbi.nlm.nih.gov/pubmed/22885261

11. Martínez-Bello D, López-Quílez A, Prieto AT. Spatiotemporal modeling of relative risk of dengue disease in Colombia. Stoch Environ Res Risk Assess. 2018;32: 1587–1601. doi:10.1007/s00477-017-1461-5

12. Nakhapakorn K, Tripathi N. An information value based analysis of physical and climatic factors affecting dengue fever and dengue haemorrhagic fever incidence. Int J Health Geogr. 2005;4: 13. doi:10.1186/1476-072X-4-13

13. Mordecai EA, Cohen JM, Evans M V., Gudapati P, Johnson LR, Lippi CA, et al. Detecting the impact of temperature on transmission of Zika, dengue, and chikungunya using mechanistic models. Althouse B, editor. PLoS Negl Trop Dis. 2017;11: e0005568. doi:10.1371/journal.pntd.0005568

14. Vaidya A, Bravo-Salgado AD, Mikler AR. Modeling climate-dependent population dynamics of mosquitoes to guide public health policies. Proc 5th ACM Conf Bioinformatics, Comput Biol Heal Informatics - BCB ’14. 2014; 380–389. doi:10.1145/2649387.2649415

15. Benedum CM, Seidahmed OME, Eltahir EAB, Markuzon N. Statistical modeling of the effect of rainfall flushing on dengue transmission in Singapore. Reiner RC, editor. PLoS Negl Trop Dis. 2018;12: e0006935. doi:10.1371/journal.pntd.0006935
