# Supplementary material for: Climate predicts geographic and temporal variation in mosquito-borne disease dynamics on two continents
Source: Nat Commun. 2021 Feb 23;12:1233. doi: 10.1038/s41467-021-21496-7 (PMC7902664; doi:10.1038/s41467-021-21496-7)
Supplement: Supplementary file 3 — Reporting Summary [file 41467_2021_21496_MOESM3_ESM.pdf]

nature research

Corresponding author(s): Jamie M Caldwell

Last updated by author(s): Nov 30, 2020

Reporting Summary

Nature Research wishes to improve the reproducibility of the work that we publish. This form provides structure for consistency and transparency in reporting. For further information on Nature Research policies, see Authors & Referees and the Editorial Policy Checklist.

Statistics

For all statistical analyses, confirm that the following items are present in the figure legend, table legend, main text, or Methods section.

n/a	Confirmed	
☐	☒	The exact sample size (*n*) for each experimental group/condition, given as a discrete number and unit of measurement
☐	☒	A statement on whether measurements were taken from distinct samples or whether the same sample was measured repeatedly
☐	☒	The statistical test(s) used AND whether they are one- or two-sided<br>*Only common tests should be described solely by name; describe more complex techniques in the Methods section.*
☐	☒	A description of all covariates tested
☐	☒	A description of any assumptions or corrections, such as tests of normality and adjustment for multiple comparisons
☐	☒	A full description of the statistical parameters including central tendency (e.g. means) or other basic estimates (e.g. regression coefficient) AND variation (e.g. standard deviation) or associated estimates of uncertainty (e.g. confidence intervals)
☐	☒	For null hypothesis testing, the test statistic (e.g. *F*, *t*, *r*) with confidence intervals, effect sizes, degrees of freedom and *P* value noted<br>*Give P values as exact values whenever suitable.*
☒	☐	For Bayesian analysis, information on the choice of priors and Markov chain Monte Carlo settings
☒	☐	For hierarchical and complex designs, identification of the appropriate level for tests and full reporting of outcomes
☐	☒	Estimates of effect sizes (e.g. Cohen's *d*, Pearson's *r*), indicating how they were calculated

Our web collection on statistics for biologists contains articles on many of the points above.

Software and code

Policy information about availability of computer code

Data collection	No software was used for data collection.
Data analysis	R statistical software version 3.5.3 was used for all analyses. R code is available at https://github.com/jms5151/SEI-SEIR_Arboviruses. The deSolve and stats R packages were used. ArcGIS v 10.4.1 was also used to calculate the population size around Kenya study sites.

For manuscripts utilizing custom algorithms or software that are central to the research but not yet described in published literature, software must be made available to editors/reviewers. We strongly encourage code deposition in a community repository (e.g. GitHub). See the Nature Research guidelines for submitting code & software for further information.

Data

Policy information about availability of data

All manuscripts must include a data availability statement. This statement should provide the following information, where applicable:

- Accession codes, unique identifiers, or web links for publicly available datasets
- A list of figures that have associated raw data
- A description of any restrictions on data availability

All data that support the findings of this study are deposited in a public repository, with the exception of arboviral incidence data from Ecuador, which is available from the corresponding author upon reasonable request. Climate data, epidemic characteristics, socio-economic, vector, and Kenya arboviral incidence data that we analyzed in this study are available in the following public repository: https://github.com/jms5151/SEI-SEIR_Arboviruses. This data supported figures 3, 4, and 6 in the main text and supplemental figures 1-3 and 6-10. Data that supports figure 7 is available in supplemental table 1. The arboviral case data from Ecuador are available from the corresponding author upon reasonable request. The data are not publicly available due to a Confidentiality Agreement with the Ecuador Ministry of Health. Crude birth and death rates used in the model are from The World Bank Open Data. The URL for crude birth rate in Ecuador is https://data.worldbank.org/indicator/SP.DYN.CBRT.IN?locations=EC and for Kenya is https://data.worldbank.org/indicator/SP.DYN.CBRT.IN?locations=KE. The URL for crude death rate for Ecuador is https://data.worldbank.org/indicator/SP.DYN.CDRT.IN?locations=EC and for Kenya is https://data.worldbank.org/indicator/SP.DYN.CDRT.IN?locations=KE. NOAA Global Surface Summary of the Day data

is available at https://www.ncei.noaa.gov/access/metadata/landing-page/bin/iso?id=gov.noaa.ncdc:C00516. NOAA Climate Prediction Center Africa Rainfall Climatology dataset is available at https://www.cpc.ncep.noaa.gov/products/african_desk/cpc_intl/cf_test/africa/arc/arc_run_wa.shtml.

Field-specific reporting

Please select the one below that is the best fit for your research. If you are not sure, read the appropriate sections before making your selection.

☐ Life sciences ☐ Behavioural & social sciences ☒ Ecological, evolutionary & environmental sciences

For a reference copy of the document with all sections, see nature.com/documents/nr-reporting-summary-flat.pdf

Ecological, evolutionary & environmental sciences study design

All studies must disclose on these points even when the disclosure is negative.

Study description	This study uses a dynamic deterministic SEI-SEIR model and validates the model output with mean monthly mosquito abundance and arboviral incidence data from eight sites in Ecuador and Kenya. The study compares the model output with observed data using pairwise correlation with an adjusted p-value using the Modified Chelton Method to account for autocorrelation and linear regressions. Comparisons between pairwise correlation and socio-ecological factors also use linear regressions.
Research sample	The research sample is Aedes aegpyti mosquitoes and human cases of dengue, chikungunya, and Zika aggregated by month, year, and site. Aedes aegypti mosquitoes were chosen because they are the primary vector for dengue, chikungunya, and Zika. The population samples are meant to represent subsamples of the mosquito population through time and disease incidence through time in different locations.
Sampling strategy	Mosquitoes were collected once or twice a month, depending on the region, and across multiple houses per site over multiple years. The data was aggregated by month, year, and site. In Ecuador, the sample size was based on preliminary data from a dengue surveillance survey, which suggests that one third of households will produce female Aedes aegypti (Stewart-Ibarra et al., unpubl.). Using that information, we estimated that of 1,800 samples (150 households * 4-month dengue season * 3 sites), approximately 600 would yield female Aedes aegypti. In Kenya, the sample size was determined by delineating the study sites into zones across environmental habitat strata and selecting a few houses within each zone. In both Ecuador and Kenya, we attempted to include houses that were farther apart than the flight distance of Aedes aegypti (~150m).<br><br>For arboviral incidence data in Kenya, sample sizes were based on prior studies. We conducted a power analysis based on prior arboviral incidence data in the Kenya study sites and used a conservative estimate of 1/50 to estimate that a sample size in the range of 1,478 to 1,890 pairs per strata (coastal or inland) will have 80% power to detect a difference in disease incidence rates of 1% using McNemar's test of equality of paired proportions with a 0.05 significance level. In contrast, we obtained the arboviral incidence data in Ecuador from the Ministry of Health (MoH), which collects reported data on confirmed dengue from clinics continuously. Therefore, the sample size is based on all data collected by the MoH over the study period (period based on mosquito sampling) in the study sites.
Data collection	Local entomologists collected mosquito data in each region and classified each mosquito by species, sex, and life stage. Local health officers collected human blood samples for disease testing. Blood samples from Kenya were analyzed in Kenya and at Stanford University School of Medicine. Blood samples from Ecuador were analyzed at the Ecuador Ministry of Health. All data was collected for other studies and followed appropriate protocol.
Timing and spatial scale	Mosquito data was collected monthly in Kenya between January 2014 and October 2018. Mosquito data was collected in Ecuador every one-to-two weeks during three, fourth month sampling periods between July 2016 and August 2018. The Ecuador collection was structured to capture different parts of the transmission season. As arboviruses are transmitted year-round in Kenya, mosquitoes were collected every month over the study period. Human disease cases were collected continuously between January 2014 and October 2018 from health clinics in each site.
Data exclusions	No data was excluded from the analysis.
Reproducibility	Climate, socio-ecological, vector, and some arbovirus data and code are provided for reproducibility.
Randomization	Samples were aggregated by month, year, and site.
Blinding	Blinding is not relevant to the present study as an intervention/risk factors were not tested. The present study does not have exposed/unexposed groupings nor a case status for participants.

Did the study involve field work? ☒ Yes ☐ No

Field work, collection and transport

Field conditions	Table 1 in the main text summarizes field conditions with information on geography, climate, and socio-demographics.
Location	The sampling occurred at four sites in Ecuador (Huaquillas, Machala, Portovelo, Zaruma; Longitude range = [-80.2,-79.6], Latitude

Location	range = [-3.7,-3.2], Elevation range = [6,1155]) and four sites in Kenya (Chulaimbo, Kisumu, Msambweni, and Ukunda; Longitude range = [34.6,39.7], Latitude range = [-4.3,-0.03], Elevation range = [4,1328]).
Access and import/export	A subsample of blood samples were exported from Kenya to the United States for processing, while all other samples were processed in Ecuador or Kenya. The blood sample export was in compliance with all local, national, and international laws. Permit: CDC PHS #20200506-1579A.
Disturbance	This study did not cause disturbances.

Reporting for specific materials, systems and methods

We require information from authors about some types of materials, experimental systems and methods used in many studies. Here, indicate whether each material, system or method listed is relevant to your study. If you are not sure if a list item applies to your research, read the appropriate section before selecting a response.

Materials & experimental systems

n/a	Involved in the study
☐	☒ Antibodies
☒	☐ Eukaryotic cell lines
☒	☐ Palaeontology
☒	☐ Animals and other organisms
☐	☒ Human research participants
☒	☐ Clinical data

Methods

n/a	Involved in the study
☒	☐ ChIP-seq
☒	☐ Flow cytometry
☒	☐ MRI-based neuroimaging

Antibodies

Antibodies used	IgG, IgM
Validation	Validation of this specific goat anti-human IgG antibody was performed by the manufacturer, wherein heavy chain binding was confirmed, and binding to human IgM or IgA antibodies, or other non-Ig serum proteins was not observed. Concerns of cross-reactivity with other species was not relevant, as the primary antibody target in this assay was human serum diluted in a neutral buffer.

Human research participants

Policy information about studies involving human research participants

Population characteristics	Samples from Ecuador represent a random sub-sample of febrile cases confirmed to have arboviral infections. Samples from Kenya represent a subsample of children less than 18 years old. We did not assess any population characteristics of the study participants.
Recruitment	Participants were included in the Ecuador Ministry of Health data if they were confirmed to have arboviral infections and participants were recruited by a passive surveillance study in Kenya.
Ethics oversight	The Ecuador Ministry of Health and the Kenya Medical Research Institute and the Institutional Review Board of Stanford University approved the protocols in Ecuador and Kenya, respectively. Permits: KEMRI ERC #2611, Stanford University IRB #31488.

Note that full information on the approval of the study protocol must also be provided in the manuscript.
